# Carbon Metabolism of a Soilborne Mn(II)-Oxidizing *Escherichia coli* Isolate Implicated as a Pronounced Modulator of Bacterial Mn Oxidation

**DOI:** 10.3390/ijms23115951

**Published:** 2022-05-25

**Authors:** Tong Gu, Zhenghu Tong, Xue Zhang, Zhiyong Wang, Zhen Zhang, Tzann-Shun Hwang, Lin Li

**Affiliations:** 1 State Key Laboratory of Agricultural Microbiology, College of Life Science and Technology, Huazhong Agricultural University, Wuhan 430070, China; gutong1117@webmail.hzau.edu.cn (T.G.); tong123@webmail.hzau.edu.cn (Z.T.); 2019zx@webmail.hzau.edu.cn (X.Z.); wangzhiyong@hbmzu.edu.cn (Z.W.); zhzh217@163.com (Z.Z.); 2 Key Laboratory of Biological Resources Protection and Utilization of Hubei Province, Hubei Minzu University, Enshi 445000, China; 3 College of Food and Bioengineering, Henan University of Animal Husbandry and Economy, Zhengzhou 450046, China; 4 Graduate Institute of Biotechnology, Chinese Culture University, Taipei 11114, Taiwan

**Keywords:** manganese oxidizing bacteria, manganese oxidation, carbon metabolism, manganese oxide, ROS

## Abstract

Mn(II)-oxidizing microorganisms are generally considered the primary driving forces in the biological formation of Mn oxides. However, the mechanistic elucidation of the actuation and regulation of Mn oxidation in soilborne bacteria remains elusive. Here, we performed joint multiple gene-knockout analyses and comparative morphological and physiological determinations to characterize the influence of carbon metabolism on the Mn oxide deposit amount (MnODA) and the Mn oxide formation of a soilborne bacterium, *Escherichia coli* MB266. Different carbon source substances exhibited significantly varied effects on the MnODA of MB266. A total of 16 carbon metabolism-related genes with significant variant expression levels under Mn supplementation conditions were knocked out in the MB266 genome accordingly, but only little effect on the MnODA of each mutant strain was accounted for. However, a simultaneous four-gene-knockout mutant (namely, MB801) showed an overall remarkable MnODA reduction and an initially delayed Mn oxide formation compared with the wild-type MB266. The assays using scanning/transmission electron microscopy verified that MB801 exhibited not only a delayed Mn-oxide aggregate processing, but also relatively smaller microspherical agglomerations, and presented flocculent deposit Mn oxides compared with normal fibrous and crystalline Mn oxides formed by MB266. Moreover, the Mn oxide aggregate formation was highly related to the intracellular ROS level. Thus, this study demonstrates that carbon metabolism acts as a pronounced modulator of MnODA in MB266, which will provide new insights into the occurrence of Mn oxidation and Mn oxide formation by soilborne bacteria in habitats where Mn(II) naturally occurs.

## 1. Introduction

Manganese (Mn) is one of the most abundant transition metal elements in the earth’s crust. In the natural environment, there are seven different oxidation states ranging from 0 to +7, among which Mn(II), Mn(III) and Mn(IV) are dominant species. The abiotic chemogenesis of Mn(II) oxide mineralization is a slow thermodynamic process under natural conditions [1], while biogenic Mn oxidation is generally 4–5 orders of magnitude faster than chemogenesis under similar conditions [2]. Therefore, natural Mn oxides are thought to be mainly derived from biological processes (BioMnOx) [2,3]. Among various Mn(II)-oxidizing microorganisms, bacteria are considered to be a vital driving force in the biological formation of Mn oxides that can quickly oxidize Mn(II) to Mn(III) or Mn(IV) on the surface of bacterial cells through various oxidases [4,5].

The naturally formed Mn oxides have been recognized as a type of strong oxide in the natural environment and are capable of potentially acting as an electron acceptor for anaerobic respiration. It has long been hypothesized that Mn(II) oxidation could be thermodynamically beneficial for diverse autotrophic bacteria in terms of the energy utilization [1,6], which was confirmed by a recent finding that the existence of chemolithoautotrophic bacteria capable of Mn(II) oxidation in an enrichment coculture of two bacterial species [7], including a major species that is affiliated to the phylum Nitrospirae but is distantly related to known species of *Nitrospira* and *Leptospirillum*, and a minor bacterium that belongs to β-Proteobacteria, namely, *Ramlibacter lithotrophicus*, which does not oxidize Mn(II) alone [7]. The two bacterial species have been confirmed to be able to use Mn(II) oxidation for CO_2_ fixation and possibly produce energy substances through the reverse tricarboxylic acid (TCA) cycle [7]. In addition, some heterotrophic bacteria isolated from the aerobic/anoxic ocean interface, such as the *Aurantimonas* sp. strain SI85-9A1, have been shown to be capable of oxidizing Mn(II) to Mn(III/IV) oxides during heterotrophic growth [8,9]. Although the electron acceptor and reaction condition requirements are still unknown, the cells appeared to be able to use Mn(II), reduced sulfur species and carbon monoxide for energy supplementation to accelerate heterotrophic efficiency and perform carbon fixation through the Calvin cycle [10].

Although the details of Mn(II) oxidation catalyzed by bacteria have continued to expand [11,12,13], the physiological roles of those activities in host cells generally remain unclear [14,15]. As a trace element, many cell functions require the participation of Mn [16,17,18]. Regardless of whether it is from the perspective of protecting itself from external environmental interference or storing excess electrons, there are multiple potential benefits of Mn(II) oxidation. For example, some bacteria could employ Mn oxide as an extracellular crust to protect against predation or as a flexible mechanism to attenuate intracellular reactive oxygen species (ROS) [19,20,21]. In addition, several previous investigations have proposed that the physiological function of manganese is closely associated with energy acquisition, such as carbon metabolism [22,23], which is consistent with that of recently revealed chemoautotrophic Mn(II)-oxidizing bacteria [7]. However, for many nonautotrophic bacteria relative to Mn metabolism, although some of them have been identified to gain a competitive growth advantage over other microorganisms [24] or to thrive in stressful environments [25] by maintaining optimal Mn levels, the correlation between Mn metabolism and other intracellular substance and energy metabolism remains elusive.

In a previous investigation, we confirmed that Mn oxides produced by a soilborne *Escherichia coli* MB266 with a pronounced Mn(II)-oxidizing activity (MnOA) by 65 μmol/L were mainly deposited on the cell surface, gradually forming Mn(III)-dominated flocculent Mn oxides, which were ultimately oxidized to form needle and crystal configurations, while small amounts of oxides were released into the surrounding environment of the cells [26]. These Mn oxide-deposited cells served as nucleation centers for the ultimate formation of Mn oxide micronodules and Mn-rich aggregates. This cell-surface-actuated Mn oxidation and Mn oxide aggregates formation usually required a relatively long Mn oxidation duration [26]. Therefore, the Mn oxide deposit amount (MnODA) formed by MB266 cells depended on both Mn oxide formation rate and nucleation processing by adsorption. The resorption-based Mn oxide aggregate processing was also confirmed in other Mn(II)-oxidizing bacteria [27,28,29], which reflects the naturally occurring Mn oxide mineralization by Mn(II)-oxidizing bacteria [30]. To date, the bacteria that utilize Mn oxidation for nutrient metabolism are usually identified at the anaerobic/aerobic oceanic interface [31]; the relationship between carbon metabolism and Mn oxidation in soilborne bacteria are still scarcely investigated.

In the current study, we performed in-depth joint analyses on the influence of carbon metabolism on the MnODA and Mn oxide configuration formed by MB266 based on multiple gene knockout using clustered regularly interspaced short palindromic repeats (CRISPR) technology and multilayered physiological and morphological assays. A previously performed proteomics analysis on this bacterium revealed that carbon metabolism genes were potentially associated with Mn(II) oxidation [32]. A total of 16 carbon metabolism-related genes with varying expression levels upon Mn supplementation culture conditions were selected for knockout and then a comparative determination of the MnODA of each corresponding gene-disruptive mutant strain was performed. We then constructed a mutant strain named MB801 with the simultaneous knockout of four genes to characterize the effects of different metabolic pathways on MnODA and performed analyses on the correlation of the intracellular ROS level with MnODA, as well as the effects of these metabolic pathways on the Mn oxide formation rate and the aggregate morphological patterns to provide new insights into the relationship between carbon metabolism and MnODA-Mn mineralization in soilborne bacteria.

## 2. Results

### 2.1. Effects of Different Carbon Source Materials on the MnOA of E. coli MB266

**Figure 1:**
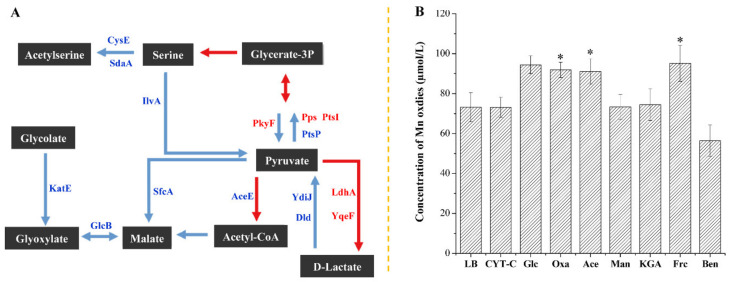
Carbon metabolic pathways potentially associated with Mn oxidation by proteomics analysis (**A**) and the effects of different carbon source substrates on the MnODA of *E. coli* MB266 suspension (**B**). Red/blue arrows in A indicate significantly upregulated/downregulated metabolic reactions, respectively. Abbreviations are listed as follows: CysE, Serine acetyltransferase; SdaA, L-Serine deaminase I; IlvA, Threonine deaminase; PkyF, Pyruvate kinase I; Pps, Phosphoenolpyruvate synthase; PtsI, PEP-protein phosphotransferase; PtsP, Fused PEP-protein phosphotransferase (enzyme I); KatE, Hydroperoxidase HPII (III); SfcA, Malate dehydrogenase; AceE, Pyruvate dehydrogenase; Ydij, FAD-linked oxidoreductase; Dld, a NADH independent D-lactate dehydrogenase; LdhA, D-Lactate dehydrogenase; YqeA, Acyltransferase; GlcB, Malate synthase G. In B, MB266 cells were cultured in LB broth with different additionally added carbon source substances and 5 mmol/L MnCl_2_ for 120 h at 37 °C, then MnOA was determined accordingly. The dose used and abbreviations are listed as follows: LB, 0 addition; CYT-C, 10 µmol/L cytochrome C; Glc, 10 mmol/L glucose; Oxa, 10 mmol/L sodium oxalate; Ace, 10 mmol/L sodium acetate; Man, 10 mmol/L mannose; KGA, 10 mmol/L α-ketoglutarate; Frc, 10 mmol/L fructose; Ben, 10 mmol/L sodium benzoate. No detectable MnODA was recorded in each negative control (without inoculation of MB266 cells) after 30 days of incubation. Means followed by * in a column were significantly different (*n* = at least 3; *p* < 0.05) according to Fisher’s protected LSD test.

We previously performed a quantitative proteomics analysis on the Mn(II)-oxidizing *E. coli* MB266 strain [32] and revealed that a variety of carbon metabolism-related genes were significantly varied in expression levels following the supplementation with Mn during culture processing (Figure 1A), thus indicating that these genes could be potentially involved in Mn oxidation. To determine whether certain carbon sources play a critical role in MnODA, MB266 cells were subjected to a continuous 120 h culturing course under the supplement of Mn(II) and different carbon source substances in LB broth, and the accumulative Mn oxides (MnODA) in the cell suspension were quantified by the standard leucoberbelin blue (LBB) assay in 120 h [33]. As shown in Figure 1B, the effects of various carbon source substances on the formed MnODA varied remarkably; for example, a notable increase was observed with acetate (Ace) by ~24%, sodium oxalate (Oxa) by ~26%, glucose (Glu) by 29% and fructose (Frc) by ~30%; a slight increase was observed with mannose (Man) and ketoglutarate (Kga) by less than 1%, and a decrease was observed with sodium benzoate (Ben) by ~23%. Not surprisingly, cytochrome c (CYT-C) did not exert an influence on Mn oxide since it is not a real carbon source material. These results demonstrate that some carbon source materials preferably fuel the formation of Mn oxides in MB266 cells, which implies that carbon metabolism in MB266 cells could modulate Mn oxidation in principle.

### 2.2. Different Metabolic Pathways Have Dissimilar Promoting Effects on the MnOA

**Figure 2:**
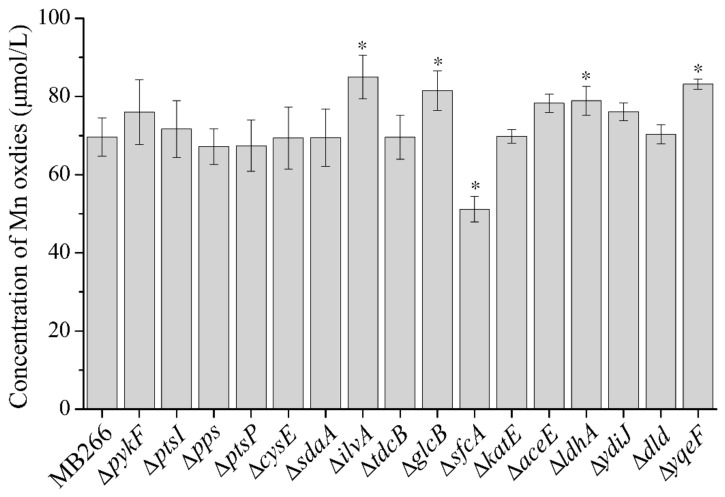
Measurement of MnODA of cell suspension in various gene-knockout mutant strains. The cells were cultured for 5 days under the supplement of 5 mmol/L MnCl_2_ at the final concentration prior to the standard LBB assay. Means followed by * in a column were significantly different (*n* = at least 3; *p* < 0.05).

**Figure 3:**
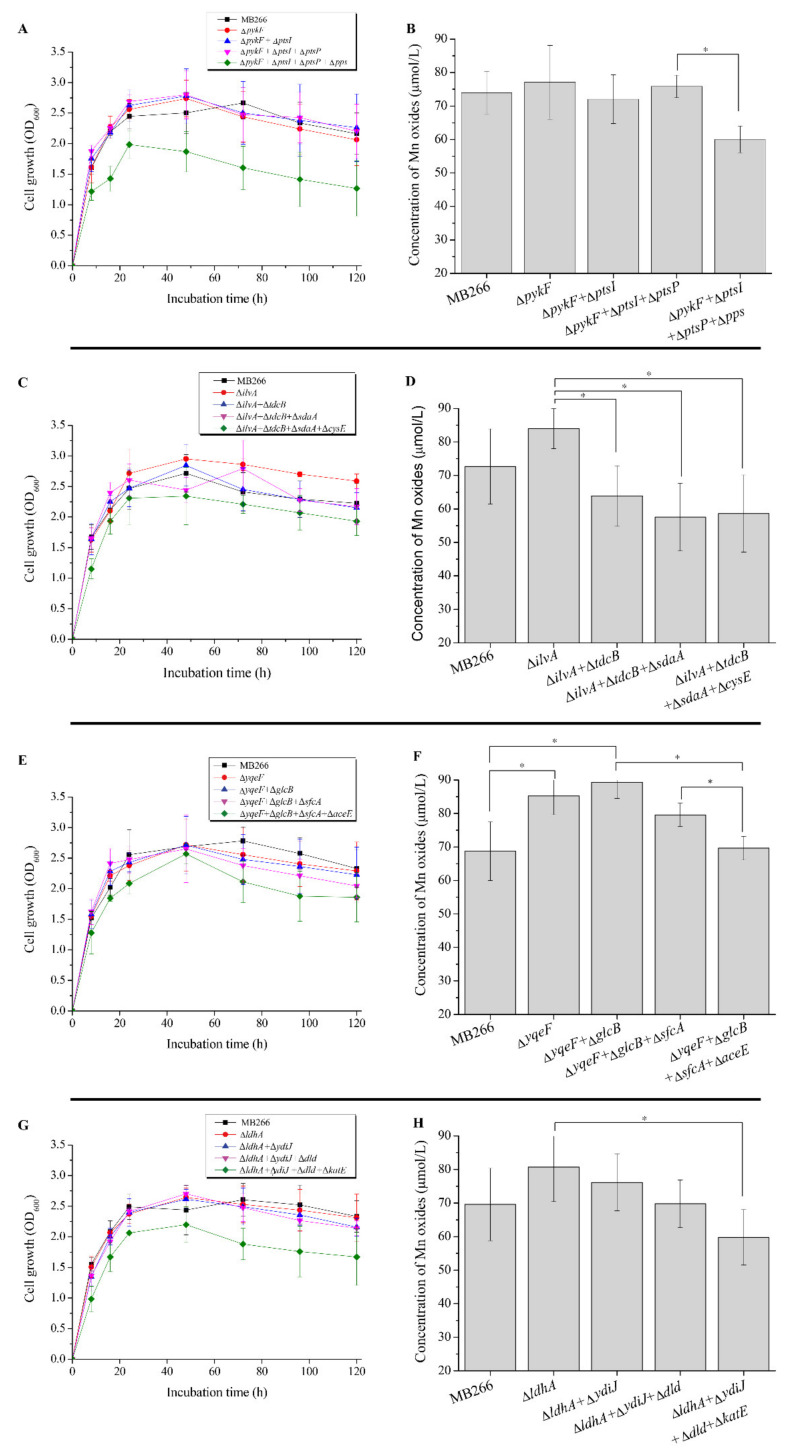
Measurements of the growth curve and MnODA of various gene-knockout mutant strains. (**A**,**C**,**E**,**G**) Growth curve; (**B**,**D**,**F**,**H**) MnODA (refers to concentration of Mn oxides) of (**A**,**C**,**E**,**G**). (**A**,**B**), pyruvate metabolic pathway; (**C**,**D**), glucogenic amino acid metabolic pathway; (**E**,**F**), glyoxylic acid metabolic pathway; (**G**,**H**), lactic acid metabolic pathway. The wild-type strain MB266 was used as the control for all measurements. Means followed by * in a column were significantly different (*n* = at least 3; *p* < 0.05).

Based on the proteomics analysis, a total of 16 carbon metabolism-related genes in MB266 (Table 1) with significantly varied expression levels following a continuous culture time course under Mn supplementation were selected as target genes to verify their involvement in the Mn oxidation of MB266. Therefore, these genes were knocked out using a modified CRISPR–Cas9 gene-editing system (Supplementary Figure S1) to yield each corresponding gene-disruptive mutant strain (Supplementary Table S1). Subsequently, the MnODA (refers to the concentration of Mn oxides of cell suspension) of each mutant strain was determined following the standard LBB assay. Figure 2 shows the diverse effects of the disrupted genes on the MnODA of MB266 cell suspensions. In the positively MnODA-promoting mutants, the top three mutants were Δ*ilvA*, Δ*yqeF* and Δ*glcB* (for the corresponding proteins, see Table 1), and compared to the wild-type MB266, they promoted MnODA by approximately 22.0%, 19.4% and 18.2%, respectively. The other positive mutants included Δ*ldhA*, Δ*aceE*, Δ*ydiJ* and Δ*pykF*, which increased the MnODA from approximately 13.4% to 9.2%. Meanwhile, a significant reduction in the MnODA occurred in Δ*sfcA* by approximately 26.5%, and the disruption of other genes only exhibited a limited effect on MnODA. However, interestingly, it appeared that all of the MnODA-increased mutants were derived from those repressing certain anaerobic fermentation pathways, i.e., IlvA [34] is a threonine dehydratase that can be activated and increased in anaerobic cultured cells; GlcB [35], YqeF [36] and AceE [37] participate in the process of glyoxylic acid metabolism, while LdhA [38] and YdiJ [39] are catalytic enzymes of lactic acid metabolism. Thus, we speculate that the diverse effects of different carbon metabolism-related genes on MnODA might be the consequence of altered metabolic pathways rather than the gene alone. Therefore, we performed further continuous progressive multigene knockout in four carbon metabolic pathways of pyruvate alone (Figure 3A), glucogenic amino acids (Figure 3C), glyoxylic acid (Figure 3E), lactic acid (Figure 3G) and comparative MnODA assays. As shown in Figure 3A,C,E,G, each increased gene knockout count caused a parallel decline in the growth rate of the cells in all four carbon metabolic pathways, suggesting that these genes exerted coordinative and integral effects on metabolism. Notably, an overall consistent pattern between cell growth and MnODA was observed in all four carbon metabolic pathways, as the changes in the cell growth rate in each gene-knockout mutant of each metabolic pathway were generally followed by coordinated changes in MnODA, i.e., following the increased gene-knockout count, the cell growth rate was increasingly reduced in each carbon metabolic pathway (Figure 3A,C,E,G) and caused a synchronous reduction in MnODA (Figure 3B,D,F,H), indicating that certain carbon metabolism pathways were able to modulate MnODA by affecting the growth rate of the cells. Notably, while cell growth was increasingly repressed by the increased gene-knockout count to a certain threshold number, the varying trend of MnODA appeared to be dissimilar in different metabolic pathways, as the gene-knockout mutations in glucogenic amino acid metabolism (Figure 3D) exerted a gradual decreasing trend of MnODA most of the time, whereas the mutations in pyruvate metabolism (Figure 3B) caused an overall promotion effect on MnODA. Moreover, the knockout of certain genes in glycogen amino acid metabolism (Figure 3D), glyoxylic acid metabolism (Figure 3F) and lactic acid metabolism (Figure 3H) caused an initial promotion of MnODA, which then decreased to a relatively low MnODA compared with the wild-type MB266. Therefore, these results verify in principle that different carbon metabolism pathways have different modulation effects on MnODA.

### 2.3. Effect of Simultaneous Knockout of Several Carbon Metabolic Genes on Mn Oxide Formation

**Figure 4:**
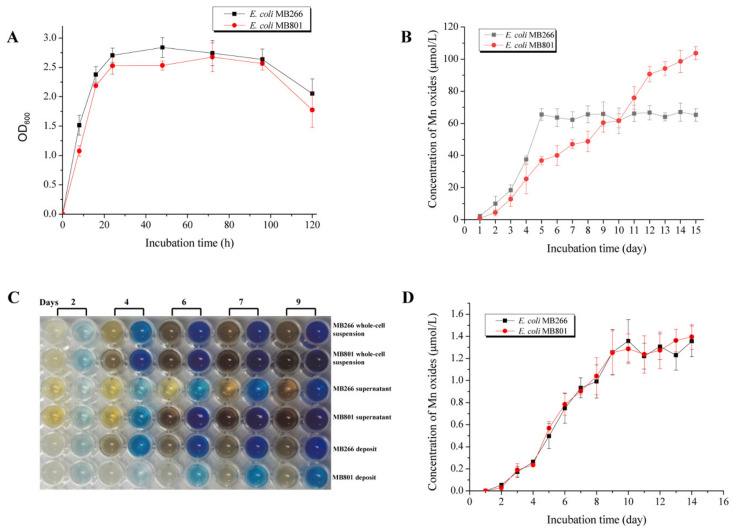
Growth curve (**A**) and MnODA (**B**,**D**) measurements and LBB test (**C**) on culture fractions of the mutant strain MB801. In (**A**), the wild-type MB266 was used as the control. The OD_600_ values were comparatively determined based on the approximately equivalent inoculum size of both strains. In (**B**), MnODA (refers to concentration of Mn oxides) of the deposit fraction of the MB801 culture was determined following the culturing of the cells for 5 days in LB broth containing 5 mmol/L MnCl_2_ at the final concentration. In (**C**), LBB test results are shown for the culture fractions of MB801 in 96-cell microtiter plates after culturing for 5 days in LB broth containing 5 mmol/L MnCl_2_ at the final concentration. In (**D**), MnODA (refers to concentration of Mn oxides) was determined over a culture time course of 5 days in Lept medium containing 100 μmol/L Mn(III) complex at the final concentration.

To further investigate the effective genes of the carbon metabolism pathway that are responsible for MnODA promotion without radically altering the growth rate of the cells, we minimized the selected mutant genes contributing the most to MnODA promotion by four in different carbon metabolic pathways, i.e., *ilvA* (glucogenic amino acid metabolism), *glcB* (glyoxylic acid metabolism), *ldhA* (lactic acid metabolism) and *yqeF* (glyoxylic acid metabolism), which each alone exhibited the most promoting MnODA in its corresponding pathway, to construct the recombinant *E. coli* MB801 using the CRISPR–Cas9 gene-editing system by knocking out these genes simultaneously. We then determined the MnODA of different cell fractions following continuous cell culturing for 15 days. Figure 4A shows that the mutant strain MB801 maintained a uniform growth pattern with the wild-type MB266, with a nearly identical growth rate and cell density during the culture time course. However, the MnODA of the culture precipitate of MB801 and MB266 exhibited a different development trend (Figure 4B). While the MnODA in the deposit of the MB266 culture reached the peak value on the 5th day and then maintained a stable level during the rest of the culture time course, that of MB801 initially accumulated slowly but had a sharp and steadily increasing pattern over the culture time course, e.g., on Day 5, the level was approximately 56% of that of MB266, a similar level was reached on Day 10, and then the level increased by approximately 59%, relative to MB266 on Day 15. It is worth noting that although the MB801 cells showed the ultimately highest MnODA over MB266, the time to reach this highest value was significantly deferral. Interestingly, a parallel comparative measurement of the supernatant Mn oxides on both strains showed that the relatively high level of Mn oxides occurred in MB801 over MB266, while the total Mn oxides of both strains were nearly identical (Figure 4C), indicating the potentially different formation processes of insoluble Mn oxide deposits of both strains as a result of the occurrence or nonoccurrence of these genes, which elicited changed regulatory mechanisms underlying Mn oxide formation. Moreover, we further investigated whether the ultimate increase in Mn oxides in MB801 was specifically derived from the increased conversion of Mn(III) to Mn(IV). MB801 cells were cultured in LB broth containing 100 μmol/L of the Mn(III) complex. As shown in Figure 4D, both MB801 and MB266 cells had nearly synchronized Mn oxide formation patterns following the culture time course, indicating that the increase in Mn oxides in MB801 was not derived from the process of conversion from Mn(III) to Mn(IV); conversely, it was more likely to be the process of conversion from Mn(II) to Mn(III). Overall, these results confirmed that cellular carbon metabolism was deeply involved in the formation of Mn oxides, with multilayered and interactional metabolic and regulatory networks and mechanisms.

### 2.4. Comparative Characterization of Mn Oxide Aggregate Structures Formed in MB801 and MB266

**Figure 5:**
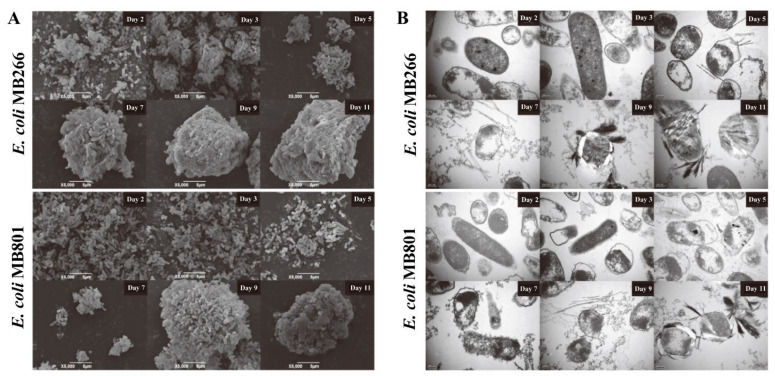
SEM (**A**) and TEM (**B**) micrographs of Mn oxide aggregate formation during the culture time course. The cells were cultured in LB broth containing 5 mmol/L MnCl_2_ at the final concentration for 11 days. In (**A**), the samples were statically suspended for 30 min at room temperature, the precipitates were collected by pipetting out the supernatants, and 2.5% glutaraldehyde was added then placed at 4 °C for 12 h, followed by sample drying with ethanol dehydration and freeze-drying processing for SEM observation. In (**B**), the samples were vigorously stirred and 2.5% glutaraldehyde was added and placed at 4 °C for 12 h.

The deposited Mn oxide agglomerates formed by the MB801 and MB266 cell suspensions were sampled at different culture time intervals for the SEM observations. As shown in Figure 5A, the SEM profiles of both strains showed that their Mn oxide formation processes exhibited a basically uniform pattern in terms of forming ultimate regular microspherical aggregates upon continuous Mn(II)-enriched culturing, and the attached and embedded bacteria around the aggregates can be easily distinguished from both strains. In MB266, some initial cells/Mn oxide agglomerates were formed on Day 3 and these cells/Mn oxide agglomerates gradually formed larger regular microspherical cells/Mn oxide aggregates from Day 7 to Day 11, with the diameters of the aggregates varying from approximately 10 μm to 20 μm. In contrast, the aggregate formation was relatively slow, with only few dispersed cells/Mn oxide agglomerates observed from Day 5 to Day 7, and the average aggregate size was also smaller in MB801. Furthermore, the degree of mineralization on the surface of the MB801 aggregate was significantly deficient relative to MB266 on Day 9.

TEM observations were performed to check the profiles of the aggregate composition cell surface coverage and structural configuration of Mn oxide deposits formed in MB266 and MB801 (Figure 5B). It was shown that the cell surface of MB266 was nearly covered by Mn oxides on Day 9, whereas the surface self-structure of some MB801 cells were still clearly observed on Day 9, indicating the synchronous deferral mineralization on the cell surface of MB801 compared to MB266 following the deferral of the agglomerate formation. It was also visualized that MB266 cells had some filamentous crystalline structural deposits of Mn oxides on their surface around the 5th day and 7th day, whereas only flocculent-like Mn oxides were deposited onto the surface of the MB801 cells. The surface of a certain percentage of MB266 cells was completely surrounded by Mn oxide crystals around the 9th day, but only filamentous crystalline Mn oxide deposits were initially formed on the surface of the MB801 cells. On the 11th day, a thick crystalline deposit layer formed on the surface of MB266 cells, strongly contrasting with the fewer crystalline Mn oxide deposits on the surface of the MB801 cells, indicating that although relatively more Mn oxide was produced by the MB801 cells, the Mn oxide deposits in a generally crystalline structural conformation were relatively fewer and had just begun to form a crystal-like structure. These results suggest that the nongrowth rate-dependent effect of carbon metabolism genes on the MnOA of the cells is implicated in the structural changes of biogenic Mn oxides.

### 2.5. Influence of Carbon Metabolism on MnODA Implicated in Intracellular ROS Levels

**Figure 6:**
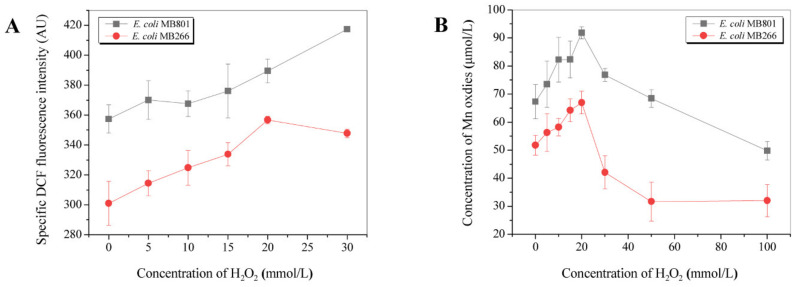
Effect of ROS on the MnODA of MB801. In (**A**), the cells were cultured for 24 h prior to the addition of H_2_O_2_ at various concentrations. The specific DCF fluorescence intensity was recorded. In (**B**), the MnODA of the cells was determined after culturing the cells for 5 days with H_2_O_2_ at different concentrations.

Several previous investigations revealed that the oxidation of soluble Mn(II) to Mn(III) was directly related to the oxidative stress level in the cells [4,20]. Therefore, we performed comparative determinations of intracellular ROS levels in MB801 and MB266 cells to investigate the effects of the ROS level on the MnODA. Figure 6A shows that the intracellular ROS level of MB801 cells cultured for 24 h was significantly higher than that of MB266 cells. Moreover, the external addition of H_2_O_2_ exhibited a varied and dramatic impact on the MnODA of both 24 h cultured strains, with a sharp increasing activity pattern observed until the threshold value of H_2_O_2_ by 20 mmol/L, where the highest MnODA of MB266 and MB801 were observed at approximately 36.6% and 29.2%, respectively; then, a rapid decrease was observed following the increased H_2_O_2_ over 20 mmol/L, with a final decrease of 26.9% and 38.2% at 100 mmol/L of H_2_O_2_ compared to that at 0 of H_2_O_2_, respectively, indicating that the MB801 cells were more susceptible to the H_2_O_2_ treatment in terms of MnODA.

**Figure 7:**
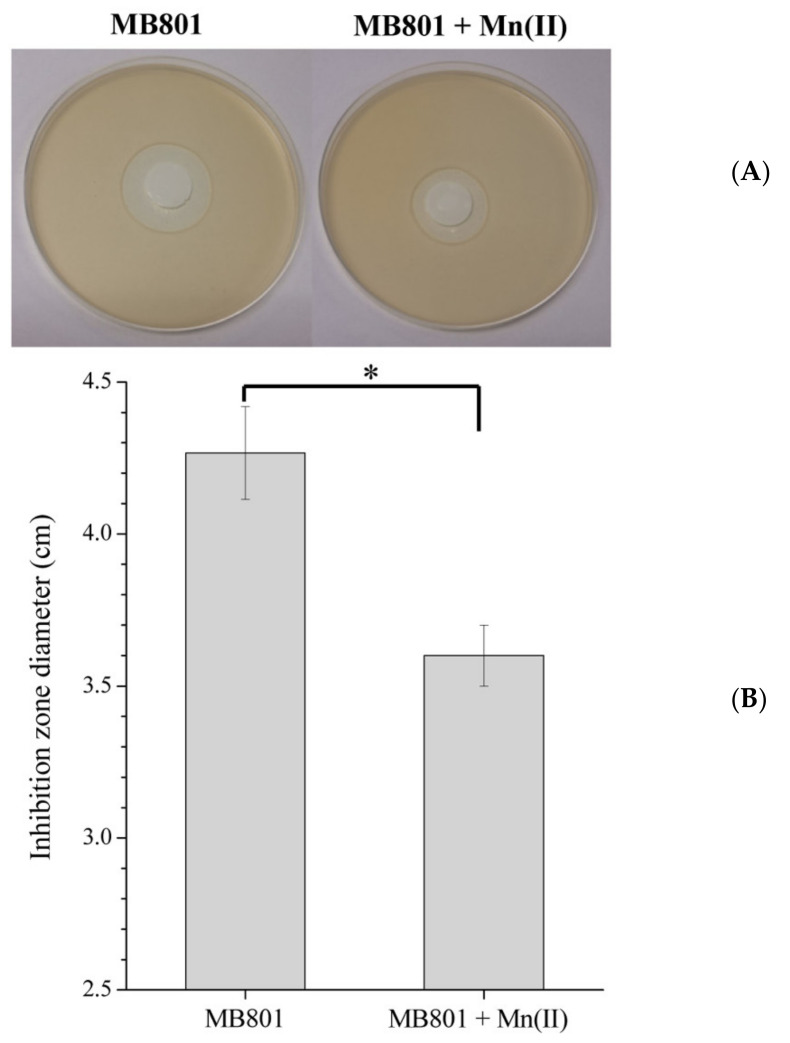
H_2_O_2_ inhibition zone on the growth of MB801. In (**A**), sterile 6-mm-diameter filter papers were immersed in 10 µL of 1 mol/L H_2_O_2_, and 5 mmol/M MnCl_2_ was used as appropriate. In (**B**), the plotted inhibition zone diameters are shown with/without MnCl_2_ supplementation. Means followed by * in a column were significantly different (*n* = at least 3; *p* < 0.05).

The growth inhibition of both strains by H_2_O_2_ was performed to verify the effect of H_2_O_2_ on cell growth. As shown in Figure 7, the H_2_O_2_ treatment caused a negative inhibition of the growth of MB801 cells because an inhibition zone was clearly visualized (Figure 7A). However, interestingly, after cultivating MB801 cells under Mn(II) supplementation for 120 h, the size of the inhibition zone by H_2_O_2_ was significantly reduced (Figure 7A,B), indicating that the process of Mn oxidation can enhance the ability of MB266 to resist intracellular and extracellular ROS.

## 3. Discussion

To date, the Mn oxidation processes driven by soilborne bacteria have been revealed to occur under diverse conditions, including some harsh environments with nutrient deficiency or the presence of toxic substances [1], although the molecular mechanisms underlying biogenic Mn oxidation biomineralization and the determinants of Mn oxidation initiation and MnOA remain elusive. We have recently reported the significant variation of certain carbon metabolism-related genes in highly reactive Mn(II)-oxidizing *E. coli* MB266 following a prolonged culturing course upon Mn(II) supplementation [32], suggesting the potential involvement of these genes in cellular Mn(II) oxidation processes. The aim of the current study was to investigate the role of these previously manifested genes in Mn(II) oxidation and whether some of them were preferable to MnODA and the formation of a specific Mn oxide configuration in laboratory trials. Although carbon source substances have generally been recognized as crucial molecular skeletons and energy substances for cell life, the specific relationship between certain carbon sources and the oxidative process of Mn(II) and Mn oxide configurations has not been reported to date. Here, we performed joint biophysiological and morphological analyses to characterize the influence of carbon sources and metabolic pathways on MnODA and Mn oxide formation. Collectively, our results clearly showed that carbon source substances and metabolic pathways significantly modulated MnODA and Mn oxide formation with multilayered and interactional networks and mechanisms, which could provide new insights into the diversified occurrence of Mn oxide mineralization and the mechanistic elucidation of the actuation of Mn(II) oxidation in natural habitats.

### 3.1. Variances in MnOA Were Highly Related to the Overall Carbon Metabolic Level of the Cells

The experiments undertook on the correlation between various carbon source substances. It is noteworthy that various metabolic pathways had different functions in the Mn oxidation capacity of the cells, while some of them promoted MnOA to different extents, and the inhibition of anaerobic fermentation profitably promoted MnOA. For instance, the effects of glyoxylic acid and lactic acid metabolism on MnOA appeared to be more significant than those of pyruvate and glucogenic amino acid metabolism in terms of MnOA. These results confirmed the promoting effect of some preferred carbon sources on the Mn oxidation of MB266 (Figure 1B). This effect may be driven by higher oxidative stress and diverse intermediate metabolites during metabolism of the preferred carbon source. However, when the changes in the carbon metabolism pathway were too drastic, a significant decrease in growth rate would wipe out all promotion effects and further lead to a reduction in MnODA (Figure 3). In addition, it is also worth noting that the effect of carbon metabolism on Mn oxidation was not only reflected in the maximum value of MnODA, but also affected the time course of reaching the maximum MnODA value (Figure 4B). Therefore, it is of interest to further investigate the cross-sectional and prospective associations between carbon substance and energy metabolism and bacterial Mn oxidation in *E. coli* MB266.

### 3.2. Carbon Metabolism Can Modulate MnODA by Affecting the Resorption of Mn Oxides and the Formation of Aggregates

For most soilborne Mn(II)-oxidizing bacteria, the initial adsorption of the surrounding Mn(II) around the cells was definitely critical for Mn oxidation in the natural environment, which enabled Mn(II) ions (and the formed Mn oxides) to bind to the surface of the cells where the oxidation reaction took place. Under normal circumstances, Mn adsorption and Mn oxidation may reach a balance. However, when the Mn oxidation capacity declined significantly, the reaction on the Mn adsorption of MB266 may not match that of Mn oxidation, resulting in the defective and abnormal formation of Mn oxide aggregates in terms of structural configuration. The observations by SEM and TEM confirmed these speculations. Compared with the wild-type MB266, the mutant strain MB801 had a deferral Mn oxide aggregate formation in the early stage (Figure 5A). At 5–9 days, the surface of MB266 began to form fiber Mn oxides that gradually formed crystalline Mn oxide configurations, whereas the surface-surrounded Mn oxides in MB801 cells occurred in relatively smaller particles and even only flocculus fabrics (Figure 5B). The TEM micrographs of Days 9–11 showed that the degree of mineralization of Mn oxide aggregates on the surface of MB801 cells was far less than that of MB266. After 11 days, a large amount of Mn oxide precipitation ultimately occurred (Figure 4B,C). The ultimately increased Mn(III)-configurated Mn oxides (Figure 4B) and deferral enlarging processing of aggregates in MB801 (Figure 5A) did not radically change the oxidation of Mn(III) to Mn(IV) (Figure 4D), suggesting that the process of oxidation from Mn(II) to Mn(III) might be reinforced following the simultaneous knockout of several carbon metabolic genes. Given that the Mn(III) oxides dominated in the early-stage formed Mn oxides in some Mn(II)-oxidizing bacteria with indirect MnOA [26,30], and no dramatic change was observed in the process of oxidizing Mn(III) to Mn(IV) in MB801 (Figure 4D), it is of particular interest to investigate whether there are specific regulatory pathways in the carbon metabolism networks, which regulate the formation of Mn oxide aggregates through the change in Mn(III) amounts and configuration; such an investigation is now our main research goal.

### 3.3. Effect of Carbon Metabolism on Mn Oxide Aggregate Formation Was Highly Related to the Intracellular ROS Level

Several previous investigations have reported that the actuation of converting Mn(II) to Mn(III) is mainly implicated in horizontally transferred intracellular oxidative pressure during indirect Mn oxidation [40,41]. In a previous study, we also revealed that the indirect Mn oxidation capability of MB266 cells could be derived from the electron transport chain on the cell membrane and was related to the level of intracellular oxidative stress [26]. Under cocultivation with different concentrations of H_2_O_2_, the enhancement of MnOA in MB266 cells was positively correlated with the increase in H_2_O_2_ concentration below a threshold value (20 mmol/L, Figure 6A); the challenge test on H_2_O_2_ also verified that MB801 cells significantly decreased their sensitivity to H_2_O_2_ upon coculture with Mn(II), indicating that the presence of Mn(II) could effectively protect bacterial cells from ROS damage (Figure 7). We speculate that the reasons for this phenomenon could be as follows: (1) Mn can not only be used as an electron donor to transfer the oxidative pressure caused by intracellular ROS to reduce the damage of DNA and protein, but could also reduce the contact probability of external oxidizing substances when it is oxidized and adsorbed onto the surface of the cells. However, when the H_2_O_2_ concentration exceeds a certain threshold, the Mn oxidation capacity of MB266 decreases instead, reflecting that ROS do not always play a positive role by promoting Mn oxidation, which could be attributed to the overall decrease in growth and metabolic intensity and the subsequent decline in MnOA (Figure 6B) caused by the enormous survival pressure from an excess of intracellular ROS. (2) Due to the relatively low Mn(II) ion concentration and relatively limited Mn oxidation efficiency, Mn oxides were relatively less competitive under high oxidative pressure conditions than superoxide dismutase and some other conventional ROS scavenging substances. Therefore, when the level of intracellular ROS continues to increase, more electron transfer may be shifted to the conventional ROS scavenging system, resulting in the inhibition of the Mn oxidation process. In fact, most naturally occurring bacteria exhibit the ability to remove or attenuate oxidative stress in cells through proteins such as superoxide dismutase [42,43]. Unlike some scavenging systems by certain proteins, such as superoxide dismutase, the high tolerance brought about by the relatively low loading capacity of the Mn oxidation process can help bacterial cells maintain a certain intracellular oxidative pressure to better confront complex environmental conditions. Therefore, this flexible response system to oxidative stress confers Mn(II)-oxidizing bacteria advantages in the process of competing for survival resources with other bacteria. This may be one of the reasons why some microorganisms preferably retain the ability to oxidize Mn(II) during the long-term evolutionary process.

## 4. Materials and Methods

### 4.1. Strains and Culture Conditions

The bacterial strains used in this study are listed in Supplementary Table S1. Briefly, the wild-type *E. coli* strain MB266 was isolated from surface soil with Fe-Mn nodules in Shangdong Province, China [26]. For the production of Mn oxides, overnight-cultured bacterial cells were inoculated into lysogeny broth (LB) [44] or Lept medium [45] supplemented with MnCl_2_ at a final concentration of 1 mmol/L at a 2% (*v*/*v*) inoculum size. The cells were cultured for 5 days to 15 days at 37 °C with routine shaking at 200 rpm unless specified. For culturing recombinant *E. coli* cells, the antibiotics ampicillin (Amp), chloramphenicol (Cm) and spectinomycin (Spc) at final concentrations of 100 μg/mL, 25 μg/mL and 50 μg mL, respectively, were used when appropriate.

### 4.2. Plasmid Construction and Transformation

All plasmids and oligonucleotide primers for polymerase chain reaction (PCR) amplification used in this work are listed in Supplementary Tables S2 and S3, respectively. Bacterial genomic or plasmid DNA was prepared using standard procedures [44]. The gold gate cloning method was used to generate a specific CRISPR array in plasmid sgRNA-bacteria as previously described [46,47]. The two newly synthesized complementary oligonucleotide fragments were annealed in T4 oligo DNA annealing buffer (10 mmol/L Tris · pH 8.0, 50 mmol/L NaCl, 1 mmol/L EDTA). A 20 μL reaction system containing 300 ng of donor plasmid DNA, 10 pmol of annealed spacer DNA, 10 U of Bsa I, 10 U of T4 polynucleotide kinase and 200 U of T4 DNA ligase (all these enzymes were purchased from New England Biolabs) in T4 DNA ligase buffer was carried out for 60 cycles at 37 °C for 5 min and 16 °C for 5 min, followed by inactivation manipulation at 80 °C for 5 min. The ligation product was then directly transformed into recipient *E. coli* Mach1-T1 using previously described procedures [48].

### 4.3. Gene Disruption

The construction of recombinant mutant strains using the CRISPR–Cas9 method [49,50] is schematically illustrated in Supplementary Figure S1. To inactivate the target genes, *E. coli* MB266 cells were initially transformed with the plasmid pCas9 (Ts)-NHEJ [48,51]. Then, the plasmid sgRNA-bacteria expressing the specific CRISPR target was used to transform the cells by electroporation. The transformed cells were plated onto LB agar plates with antibiotics Cm and Spc and cultured at 30 °C. The mutations were confirmed through PCR using cross-checked primers.

### 4.4. Analytical Assays

Cell density was measured at 600 nm with a UV–Vis spectrophotometer (DU-800 Nucleic Acids/Protein Analyzer, Beckman Coulter). Cell suspensions were adjusted to a unit optical density at 600 nm (OD_600_) of 0.8, and the cell density was recorded and calculated based to the dilution multiples.

The assays of MnODA of whole-cell suspensions or deposit fractions were performed using the standard LBB method [33] based on the quantification of Mn oxides (primarily assumed to be MnO_2_). For normalized culturing, all parent bacterial cells were adjusted to a unit OD_600_ of 0.5 and were inoculated into Lept or LB medium (when appropriate) at 1.0% (*v*/*v*) of the inoculation amount. The cells were cultured across the scheduled time course and the cell suspensions were subjected to direct quantification of the content of Mn oxides without adjusting cell density. The KMnO_4_ was used as a standard to read the absorbance at 620 nm. The concentration of MnO_2_ produced (1 mmol/L MnO_2_ corresponds to 0.4 mmol/L KMnO_4_) was defined as the concentration of Mn oxides, which refers to the MnODA value of cell suspension.

The LBB test on cell suspension loaded in 96-cell microtiter plates (Costar, Corning Incorporated, Corning, NY, USA) was performed according to a previously described method [27], where 1 mmol/L MnCl_2_ (final concentration) was added to the reaction solution. For the determination of the intracellular ROS level, cells were harvested by centrifuge at 6000× *g* r/min for 15 min, washed three times with phosphate buffered saline (PBS, pH 7.4). The cell concentration was adjusted to approximately 1 × 10^7^ cells/mL using PBS, and the cell suspension was treated with 100 μmol/L 2’,7’-dichlorodihydrofluorescein diacetate (DCFH-DA) for 1 h. The fluorescence of the formed DCF that corresponds to the intracellular ROS level was determined using a fluorescence spectrophotometer (RF-5103PC, Shimadzu, Japan) at an excitation wavelength of 485 nm and an emission wavelength of 523 nm, following the previously described DCFH-DA method [52].

### 4.5. H_2_O_2_ Inhibition Zone Measurement

The overnight cultured MB266/mutant strain cells were transferred to 50 mL of newly prepared LB medium at 1% (*v*/*v*) inoculum size. After culturing at 30 °C for 6 h, the cells were collected by centrifugation and resuspended in 0.9% NaCl saline. The cell concentration of each strain was adjusted to a unit OD_600_ of 1.0. Then, 1 mL of cell resuspension was added to 100 mL of heat-liquefied LB agar at approximately 50 °C to allow vigorous mixing and poured into a petri dish to prepare the LB agar plates. A piece of circular sterile filter paper (with a diameter of approximately 6 mm) was placed in the middle of the plate, and then 10 µL of 1 mol/L H_2_O_2_ solution was added to immerse the filter paper. After culturing at 30 °C for 24 h, the diameter of the inhibition zone produced by H_2_O_2_ treatment was measured using a Vernier caliper.

### 4.6. Scanning Electron Microscopy (SEM) and Transmission Electron Microscopy (TEM) Assays

The sample preparation, SEM and TEM assays were performed following previously described procedures [53,54].

### 4.7. Data Analysis

All data in this study are presented as the average ± standard deviation (±SD) of at least three trials in parallel. Statistical analyses were performed based on an analysis of variance (ANOVA) with the SPSS statistical software package (Version 19.0; SPSS, Inc., Chicago, IL, USA). Means were separated and compared using Fisher’s protected least significant difference (LSD) test. Graphs were prepared using Origin 11 software (Origin Lab Corp., Northampton, MA, USA).

## 5. Conclusions

In conclusion, we have confirmed that carbon metabolism serves as a pronounced modulator of MnODA and Mn oxide configuration processes in a naturally occurring highly reactive Mn(II)-oxidizing bacterial isolate. While various carbon source substances conferred varied MnODA of the cells, the modulation of carbon metabolism on the MnODA was mainly carried out by affecting the formation of Mn oxide aggregates. The effect of carbon metabolism on Mn oxide aggregate formation was highly related to the intracellular ROS level. Thus, this study provides new insights into the preferable occurrence of Mn oxidation-biomineralization by soilborne bacteria in the natural environment.

## Figures and Tables

**Table 1:** Selected carbon metabolism-related proteins with significantly changed expression levels under Mn supplementation from the proteomics data of *E. coli* MB266.

Protein (Gene)	Predicted Function/Activity	Expression Fold Change
PykF (*pykF*)	Pyruvate kinase I	1.171
PtsI (*ptsI*)	PEP-protein phosphotransferase of PTS system	1.135
Pps (*pps*)	Phosphoenolpyruvate synthase	1.11
PtsP (*ptsP*)	Fused PEP-protein phosphotransferase (enzyme I) of PTS system	0.879
CysE (*cysE*)	Serine acetyltransferase	0.824
SdaA (*sdaA*)	L-Serine deaminase I	0.907
IlvA (*ilvA*)	Threonine deaminase	0.754
TdcB (*tdcB*)	Catabolic threonine dehydratase, PLP-dependent	1.189
GlcB (*glcB*)	Malate synthase G	0.81
SfcA (*sfcA*)	Malate dehydrogenase, NAD-requiring	0.816
KatE (*katE*)	Hydroperoxidase HPII (III)	1.155
AceE (*aceE*)	Pyruvate dehydrogenase	0.872
LdhA (*ldhA*)	Fermentative D-Lactate dehydrogenase	1.173
YdiJ (*ydiJ*)	FAD-linked oxidoreductase	0.848
YqeF (*yqeF*)	Acyltransferase	1.252
Dld (*dld*)	NADH independent D-lactate dehydrogenase	0.88

## Supplementary Materials

The following supporting information can be downloaded at: https://www.mdpi.com/article/10.3390/ijms23115951/s1.

## Abbreviations

Mn	Manganese
MnODA	Mn oxide deposit amount
MnOA	Mn(II)-oxidizing activity
BioMnOx	Biogenic manganese oxides
ROS	Reactive oxygen species
TCA	Tricarboxylic acid
LBB	Leucoberbelin blue
CRISPR	Clustered regularly interspaced short palindromic repeats
Ace	Acetate
Oxa	Sodium oxalate
Glu	Glucose
Frc	Fructose
Man	Mannose
Kga	Ketoglutarate
Ben	Benzoate
CYT-C	Cytochrome c
SEM	Scanning electron microscopy
TEM	Transmission electron microscopy
