# Supplementary material for: Carbon Metabolism of a Soilborne Mn(II)-Oxidizing Escherichia coli Isolate Implicated as a Pronounced Modulator of Bacterial Mn Oxidation
Source: Int J Mol Sci. 2022 May 25;23(11):5951. doi: 10.3390/ijms23115951 (PMC9180420; doi:10.3390/ijms23115951)
Supplement: Supplementary file 1 [file ijms-23-05951-s001.zip › ijms-1669392-supplementary.pdf]

Supplementary Material

Carbon metabolism of a soilborne Mn(II)-oxidizing *Escherichia coli* isolate implicated as a pronounced modulator of bacterial Mn oxidation

Tong Gu[1], Zhenghu Tong[1], Xue Zhang[1], Zhiyong Wang[1,2], Zhen Zhang[1,3], Tzann-Shun Hwang[4,*]and Lin Li[1,*]

[1] State Key Laboratory of Agricultural Microbiology, College of Life Science and Technology, Huazhong Agricultural University, Wuhan 430070, China; gutong1117@webmail.hzau.edu.cn (T.G.); tong123@webmail.hzau.edu.cn (Z.T.); 2019zx@webmail.hzau.edu.cn(X.Z.)

[2] Key Laboratory of Biological Resources Protection and Utilization of Hubei Province, Hubei Minzu University, Enshi 445000, China; wangzhiyong@hbmzu.edu.cn

[3] College of Food and Bioengineering, Henan University of Animal Husbandry and Economy, Zhengzhou 450046, China; zhzh217@163.com

[4] Graduate Institute of Biotechnology, Chinese Culture University, Taipei 11114, Taiwan

* Correspondence: lilin@mail.hzau.edu.cn (L. L.) and hzx@faculty.pccu.edu.tw (T.-S.H.).

Supplementary Tables

Table S1. Strains used in this study

Strain	Relevant characteristic(s)[a]	Sources
Escherichia coli Mach1-T1	F^{-} φ80*(lac Z)Δm15 ΔlacX74 hsdR(rkmk+)ΔrecA1398 endA1 tonA*	Laboratory stock
Escherichia coli MB266	A highly reactive Mn(II)-oxidizing bacterial isolate	Zhang et al., 2015
Δ*pykF*	MB266 with in-frame deletion of *pykF*	This study
Δ*ptsI*	MB266 with in-frame deletion of *ptsI*	This study
Δ*pps*	MB266 with in-frame deletion of *pps*	This study
Δ*ptsP*	MB266 with in-frame deletion of *ptsP*	This study
Δ*cysE*	MB266 with in-frame deletion of *cysE*	This study
Δ*sdaA*	MB266 with in-frame deletion of *sdaA*	This study
Δ*ilvA*	MB266 with in-frame deletion of *ilvA*	This study
Δ*tdcB*	MB266 with in-frame deletion of *tdcB*	This study
Δ*glyB*	MB266 with in-frame deletion of *glcB*	This study
Δ*sfcA*	MB266 with in-frame deletion of *sfcA*	This study
Δ*yqeF*	MB266 with in-frame deletion of *yqeF*	This study
Δ*aceE*	MB266 with in-frame deletion of *aceE*	This study
Δ*ldhA*	MB266 with in-frame deletion of *ldhA*	This study
Δ*ydiJ*	MB266 with in-frame deletion of *ydiJ*	This study
Δ*lpd*	MB266 with in-frame deletion of *lpd*	This study
Δ*katE*	MB266 with in-frame deletion of *katE*	This study

Δ*pyr1*	MB266 with in-frame deletion of *pykF*	This study

Δ*pyr2*	Δ*pyr1* with in-frame deletion of *ptsI*	This study
Δ*pyr3*	Δ*pyr2* with in-frame deletion of *ptsP*	This study
Δ*pyr4*	Δ*pyr3* with in-frame deletion of *pps*	This study
Δ*gaa1*	MB266 with in-frame deletion of *ilvA*	This study
Δ*gaa2*	Δ*gaa1* with in-frame deletion of *tdcB*	This study
Δ*gaa3*	Δ*gaa2* with in-frame deletion of *sdaA*	This study
Δ*gaa4*	Δ*gaa3* with in-frame deletion of *cysE*	This study
Δ*gly1*	MB266 with in-frame deletion of *yqeF*	This study
Δ*gly2*	Δ*gly1* with in-frame deletion of *glcB*	This study
Δ*gly3*	Δ*gly2* with in-frame deletion of *sfcA*	This study
Δ*gly4*	Δ*gly3* with in-frame deletion of *aceE*	This study
Δ*lac1*	MB266 with in-frame deletion of *ldhA*	This study
Δ*lac2*	Δ*lac1* with in-frame deletion of *ydiJ*	This study
Δ*lac3*	Δ*lac2* with in-frame deletion of *dld*	This study
Δ*lac4*	Δ*lac3* with in-frame deletion of *katE*	This study
MB801	MB266 with in-frame deletion of 4 genes (*ilvA*, *glcB*, *yqeF* and *ldhA*)	This study

Table S2. Plasmids used in this study

Plasmid	Relevant characteristic (s)[a]	Sources
pCas9(ts)-NHEJ	Cm^r, a pUC19 derivative harboring *spCas9,ligD,ku,Pj23119,Rep101(ts),* 11323 bp	(Su *et al.*, 2016)
pgRNA-bacteria	Sp^r, a P15A derivative harboring *Pj23119,N20,gRNA scaffold*,3833 bp	(Su *et al.*, 2016)
sgRNA-ΔPykF	pgRNA-bacteria with target N20 squence of *pykF*	This study
sgRNA-ΔPtsI	pgRNA-bacteria with target N20 squence of *ptsI*	This study
sgRNA-ΔPps	pgRNA-bacteria with target N20 squence of *pps*	This study
sgRNA-ΔPtsP	pgRNA-bacteria with target N20 squence of *ptsP*	This study
sgRNA-ΔCysE	pgRNA-bacteria with target N20 squence of *cysE*	This study
sgRNA-ΔSdaA	pgRNA-bacteria with target N20 squence of *sdaA*	This study
sgRNA-ΔIlvA	pgRNA-bacteria with target N20 squence of *ilvA*	This study
sgRNA-ΔTdcB	pgRNA-bacteria with target N20 squence of *tdcB*	This study
sgRNA-ΔglyB	pgRNA-bacteria with target N20 squence of *glcB*	This study
sgRNA-ΔSfcA	pgRNA-bacteria with target N20 squence of *sfcA*	This study
sgRNA-ΔYqeF	pgRNA-bacteria with target N20 squence of *yqeF*	This study
sgRNA-ΔAceE	pgRNA-bacteria with target N20 squence of *aceE*	This study
sgRNA-ΔLdhA	pgRNA-bacteria with target N20 squence of *ldhA*	This study
sgRNA-ΔYdiJ	pgRNA-bacteria with target N20 squence of *ydiJ*	This study
sgRNA-ΔDld	pgRNA-bacteria with target N20 squence of *dld*	This study
sgRNA-ΔKatE	pgRNA-bacteria with target N20 squence of *katE*	This study

Table S3. Primers used in this study

Primer	Sequence(5'-3')
pykF(65)-1	ctagtatgcgtctgaacttctctca
pykF(65)-2	aaactgagagaagttcagacgcat
ptsI(59)-1	ctagttgccaaggcatcagcccagc
ptsI(59)-2	aaacgctgggctgatgccttggca
pps(76)-1	ctagtctacatcattcatgccgagt
pps(76)-2	aaacactcggcatgaatgatgtag
ptsP(365)-1	ctagtctcattcaggcgtggtgcgc
ptsP(365)-2	aaacgcgcaccacgcctgaatgag
cysE(1408)-1	ctagttaaagccgaagccagaacgc
cysE(1408)-2	aaacgcgttctggcttcggcttta
sdaA(712)-1	ctagtcctcatcttcccataccgta
sdaA(712)-2	aaactacggtatgggaagatgagg
ilvA(1185)-1	ctagtcagcactgctcttaaatatt
ilvA(1185)-2	aaacaatatttaagagcagtgctg
tdcB(1490)-1	ctagtaagcgaaacaacgactggct
tdcB(1490)-2	aaacagccagtcgttgtttcgctt
glcB(26)-1	ctagtgggaacagggctggacgctg
glcB(26)-2	aaaccagcgtccagccctgttccc
sfcA(248)-1	ctagttcgctttatatcccttacgc

sfcA(248)-2	aaacgcgtaagggatataaagcga
yqeF(637)-1	ctagtggggcgttacggacacctat
yqeF(637)-2	aaacataggtgtccgtaacgcccc
aceE(17)-1	ctagtatccgatcgaaactcgcgac
aceE(17)-2	aaacgtcgcgagtttcgatcggat
ldhA(467)-1	ctagtaaaaccgctaaaactgccaa
ldhA(467)-2	aaacttggcagttttagcggtttt
ydiJ(950)-1	ctagtttgcaagagctggagcaaca
ydiJ(950)-2	aaactgttgctccagctcttgcaa
dld(139)-1	ctagtaactcaggtcgtggtacttg
dld(139)-2	aaaccaagtaccacgacctgagtt
katE(320)-1	ctagttggaatcgtgtagtggtgac
katE(320)-2	aaacgtcaccactacacgattcca

Supplementary Figures

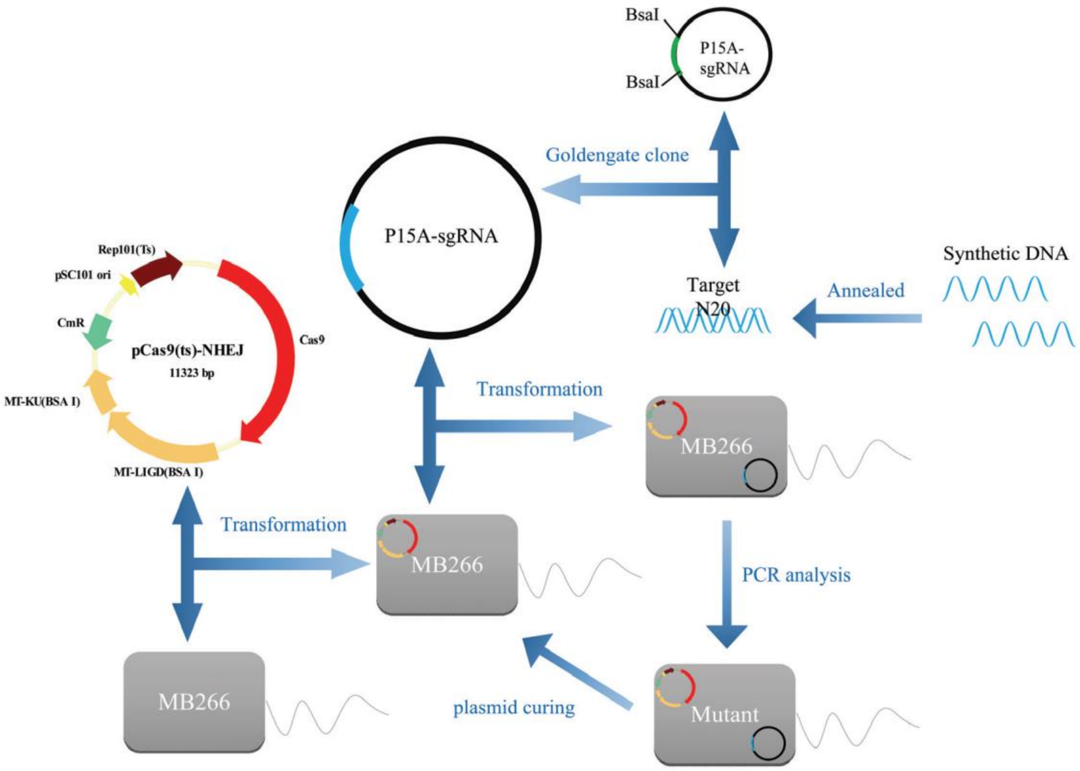

Figure S1. Schematic illustration of the inactivation of chromosomal genes by CRISPR-Cas9 in MB266. Mutagenesis is attributed to CRISPR-Cas9 system. First, the specific CRISPR array in plasmid sgRNA-bacteria was generated by the golden gate cloning method. Next, he plasmid pCas9 (Ts)-NHEJ and the plasmid sgRNA-bacteria expressing the specific CRISPR target were transformed by by electroporation. Finally, the mutations were confirmed by PCR analysis. To further engineer the strain, the sgRNA donor plasmid is cured via by culturing at 42℃ with 0.5% SDS for 24 h.
